# Iterative Development of Visual Control Systems in a Research Vivarium

**DOI:** 10.1371/journal.pone.0090076

**Published:** 2014-04-15

**Authors:** James A. Bassuk, Ida M. Washington

**Affiliations:** 1 Department of Research Continuous Performance Improvement, Seattle Children’s Research Institute, Seattle, Washington, United States of America; 2 Office of Animal Care, Seattle Children’s Research Institute, Seattle, Washington, United States of America; University of Central Florida, United States of America

## Abstract

The goal of this study was to test the hypothesis that reintroduction of Continuous Performance Improvement (CPI) methodology, a lean approach to management at Seattle Children’s (Hospital, Research Institute, Foundation), would facilitate engagement of vivarium employees in the development and sustainment of a daily management system and a work-in-process board. Such engagement was implemented through reintroduction of aspects of the Toyota Production System. Iterations of a Work-In-Process Board were generated using Shewhart’s Plan-Do-Check-Act process improvement cycle. Specific attention was given to the importance of detecting and preventing errors through assessment of the following 5 levels of quality: Level 1, customer inspects; Level 2, company inspects; Level 3, work unit inspects; Level 4, self-inspection; Level 5, mistake proofing. A functioning iteration of a Mouse Cage Work-In-Process Board was eventually established using electronic data entry, an improvement that increased the quality level from 1 to 3 while reducing wasteful steps, handoffs and queues. A visual workplace was realized via a daily management system that included a Work-In-Process Board, a problem solving board and two Heijunka boards. One Heijunka board tracked cage changing as a function of a biological kanban, which was validated via ammonia levels. A 17% reduction in cage changing frequency provided vivarium staff with additional time to support Institute researchers in their mutual goal of advancing cures for pediatric diseases. Cage washing metrics demonstrated an improvement in the flow continuum in which a traditional batch and queue push system was replaced with a supermarket-type pull system. Staff engagement during the improvement process was challenging and is discussed. The collective data indicate that the hypothesis was found to be true. The reintroduction of CPI into daily work in the vivarium is consistent with the 4P Model of the Toyota Way and selected Principles that guide implementation of the Toyota Production System.

## Introduction

Research laboratories and facilities are complex entities to manage because of the existence of a variety of challenges: human and/or animal models; experimental design and logistics; data acquisition and analysis; grant preparation and review; funding and support; publishing in the peer-reviewed literature; training of technicians, students, and fellows; infrastructure; building and engineering; and regulatory requirements. The ability to successfully manage these challenges are common targets of the principal scientist or facility director.

One way to meet these targets is to view the scientific method through the lens of continuous improvement science. Fundamental to such improvement is a high-level problem-solving algorithm known as the Plan-Do-Check-Act (PDCA) cycle, which was first reported by Shewhart in the 1930s [1] and later adopted by Deming in the 1950s [2]. The PDCA cycle has evolved into the Plan-Do-Study-Act (PDSA) cycle and has recently been reviewed [3]. Foundational to many quality improvement systems, PDCA cycles function to *(i)* realize continuous, iterative improvement, *(ii)* achieve higher quality in process results, and *(iii)* sustain continued increases in efficiency.

Rooted in the industrial production methods of the Toyota Production System (TPS) Footnote **S1**, continuous improvement science (widely referred to as lean Footnote **S2**) uses a variety of systems to help employees and managers track the completion of products in a given process. One type of system is termed visual control, which is described in the Toyota Way [6], a set of 14 Principles (Table 1) developed to support the TPS. Principle 7 teaches “to use visual control so no problems are hidden” [10], the most important step in the process of developing standardization. Attributes of visual control systems include *(i)* recognition of the information being communicated and conveyance that something about a process is abnormal, thus allowing quick action to be taken, and *(ii)* that management of relevant processes be as simple as possible. Examples of visual controls are listed in Table 2 and include the Work-In-Process (WIP) board, which is used when employees visually display their work. If an employee is assigned 10 pieces of work to complete, for example, then that employee’s WIP is equal to 10. When each piece of work is represented by a paper card, then movement along an axis of time represents progress toward completion of individual projects. When employees are assigned hundreds of pieces of work each day, then such a pitch board (Table 2) may not be practical. Instead, simple summations that are tabulated onto a visibility board can suffice and serve to alert management if *(i)* work is keeping up with demand, *(ii*) additional manpower or resources are needed to maintain progress, *(iii)* a problem needs to be addressed, or *(iv)* barriers exist that need to be removed. Visual control systems have evolved into one-screen electronic dashboards, of which the merits have recently been reviewed [11].

**Table 1:** The 14 Principles of the Toyota Way.^1^

#	Principle
1	Base management decisions on long-term philosophy at short-term sacrifice
2	Create continuous process flow in order to flush out problems
3	Develop pull systems that reduce overproduction
4	Level the workload in order to bring stability in a manner that invites standard work
5	Get quality right the first time by stopping to fix problems as they arise
6	Standardize tasks and processes in a manner that invites continuous improvement
7	Use visual controls in order to flush out problems in a manner that invites standard work
8	Use proven technology only after a clear need is thoroughly detailed
9	Grow leaders who thoroughly understand the work and enthusiastically teach it to others
10	Develop exceptional people and teams who follow the company’s philosophy
11	Challenge and help your network of partners and suppliers to constantly improve
12	Go see for yourself the actual process being performed by the actual people in the actual place
13	Make decisions by slow, studied consensus while considering all options; implement quickly
14	Become a learning organization by reflecting on learnings while continually improving

1 From reference [39].

**Table 2:** Various types of visual controls in production processes.

Type	Purpose
Alarms	A signal to alert the senses that something is not normal
Biological kanban	Based on urea levels within mouse cages, a signal for the animal care technician to transfer the mice to a clean cage – effectively removing urine and feces^1^
Charts	A visual aid that conveys inventory metrics
Colors	Used to designate how different projects are performing as a function of time
Dashboard	A visual display of the most important information needed to achieve one or more objectives that has been consolidated and arranged on a single screen so the information can be monitored at a glance [40]
Kanban	A signal for something to happen, typically a card or sign that is a means of communicating upstream precisely what is needed at the time it is needed. The kanban control card is at the heart of a pull system [5]
Heijunka board	A load-leveling board; a visual board that reflects the evening out of production in order to achieve a more consistent and even work flow [41]
Pitch board	A visual control method that serves to level the work flow; a chart that measures expected versus outcomes^2^
Reminders	A text-less signal that causes somebody to remember to do something
WIP board	A visual aid that provides knowledge of material or information that is waiting between steps in a process

1 Based on fecal and urine pattern in the dirty bedding of a mouse cage, the term *biological kanban* was originally coined by Khan and Umrysh (2008) [23]. The data presented in Results section V (Figure 5B) strengthens this term by relating ammonia levels to fecal/urine patterns – thus signaling to staff that it is time to change the cage.

2 For a more detailed discussion of pitch, and how it relates to takt time, the reader is referred to *Creating a Lean Culture* by David Mann [42].

The collective pieces of WIP are often considered to be a type of waste known as inventory. In addition to the 7 types of waste identified by Ohno [5], Liker added an 8^th^ [12] and Rampersad [13] added a 9^th^. These 9 collective types of waste are inventory, overprocessing, correction, wait time, search time, transportation, space, complexity and underutilized people [13]. Toyota controls its inventory by creating continuous process flow in order to bring problems to the surface (Principle 2) [5], [14] and by using pull systems to avoid overproduction (Principle 3) [5], [14]. Pull is defined as a method of controlling resources by replacing only what has been consumed. A pull system typically uses a signal known as a kanban, which is a trigger, or signal, to produce or replenish something. Continuous work flow is the ability of a process to demonstrate the progressive performance of tasks in a manner such that a product or service proceeds from start to consumption without stoppages. Flow defines the state of material as it moves from process to process, while pull dictates when material is moved and who (the customer) determines that it is to be moved [15]. Flow is the ultimate goal of lean, the progressive achievement of tasks so that work glides effortlessly through operations.

The visual control lessons learned by Toyota during its march to become the world’s greatest manufacturer have been adopted by healthcare. Foremost of these adopters are Wisconsin-based ThedaCare [16], [17] and two Seattle-based organizations: Virginia Mason Medical Center [18] and Seattle Children’s [19]. While the Seattle organizations differ markedly in their approach and implementation to lean Footnote S3, they both have transformed their managerial philosophy such that the patient/family is considered to be the sole customer Footnote S4.

At Seattle Children’s (Hospital, Research Institute, Foundation), the adaptation of the scientific rigor of the TPS and its 14 Principles is an organizational-wide philosophy and improvement approach called *continuous performance improvement* (CPI) [20], [21]. The Hospital’s early adopter of CPI was the clinical laboratory, which responded to increased demand by removing waste while simultaneously improving its volume and turnaround times [22]. The viability of the clinical laboratory’s approach was demonstrated by the sustainment of these gains for four years after the project’s completion. The Research Institute’s early adopter of the TPS was the Office of Animal Care (OAC), which oversees an accredited vivarium facility. Through approved animal use protocols, the OAC supports dozens of laboratories working to create cures for childhood diseases and conditions. Using CPI tools, the OAC reported that enhanced stakeholder Footnote S5 *(i.e.*, the researcher) service was a direct result of the elimination of waste, marked improvements in material flow and increased employee safety [23]. Specific improvements included a 51% reduction in cage buffer inventory, a 13% decrease in waste metrics, a 34% savings in cage wash cycle times and a 8% reduction in bottle wash cycle times. Despite initial enthusiasm [20], [24], these improvements were not able to be sustained when the vivarium relocated from temporary research housing to its permanent Research Institute home.

To address this initial setback at the Research Institute, CPI was reintroduced into the OAC via the leadership of the Research Institute’s first full-time veterinarian (I.M.W.) and an 8-month deployment of a dedicated Research CPI consultant (J.A.B). This report describes the OAC’s journey in responding to such a reintroduction and how the OAC learned that just applying a set of tools without truly understanding the complexity of the problem failed to lead to a successful improvement strategy. The long term goal of our efforts was to change the culture of the OAC from the bottom up such that each employee will *(i)* have the DNA Footnote S6 of Seattle Children’s and *(ii)* be dedicated to learning together in order to add value Footnote S7 to the patient-customer. The current report illustrates the CPI journey of the OAC in uncovering hidden work, in establishing and maturing their WIP board through the use of rapid PDCA process improvement cycles, and establishment of a biological Kanban Footnote S8 system for cage changing processes. Specific attention was paid to Principle 5: “building a culture of stopping to fix problems, to get quality right the first time”. Finally, we report our challenges that restrict the attainment of continuous work flow via pull systems.

## Materials And Methods

### I. Human Subjects

The study presented in this manuscript did not perform any research that used, created, or shared Protected Health Information. The study was therefore not subject to the State of Washington Uniform Health Information Act or the United States of America Health Insurance Portability and Accountability Act.

### II. Animal Research

At Seattle Children’s Research Institute, all animal studies are governed through protocols approved by the Institutional Animal Care and Use Committee (IACUC). The Research Institute’s Office of Animal Care (OAC) ensures that all animals used in research are treated in a humane and ethical manner, and that personnel adhere to all policies and guidelines set forth by government and private regulatory agencies. In compliance with the provisions of the Animal Welfare Act of 1966, the IACUC conducts biannual reviews of OAC institutional procedures and provides assurances to the Office of Laboratory Animal Welfare at the National Institutes of Health on a yearly basis. The animal care and use program has been fully accredited by the Association for Assessment and Accreditation of Laboratory Animal Care International since November 4, 1999.

The study presented in this manuscript did not perform any research that used animals. The study was therefore not subject to U.S. Public Law 99–158 The Health Research Extension Act of 1985 or the Animal Welfare Act of 1966.

### III. Cages, Racks and Housing Rooms

A Thoren caging system (Hazleton, PA, USA) was used to hold up to 5 mice per cage in either specific pathogen free (SPF) or regular housing rooms. Cages were contained within racks capable of high-efficiency particulate air filtration and plumbed with ultraviolet light-treated deionized water lines. Each rack contained 10 rows on the front side and 10 rows on the back side. Each row contained 6 cages. Each side therefore contained 60 cages and each rack housed 120 cages. Each SPF room was capable of housing 4 racks, or 480 cages. The SPF rooms and corridor were separated from non-SPF rooms via an airlock barrier.

### IV. Improvements via Rapid Plan-Do-Check-Act (PDCA) Process Improvement Cycles

After baseline metrics had been collected, methods for a hypothesis were developed (“Plan”) and tested (“Do”). Once improvement metrics had been captured, the results were analyzed against the hypothesis (“Check”). If the observed outcomes failed to meet expectations, then the improvement process was revised (“Act”) and retested. These cycles were repeated as many times as necessary in order to meet the target condition.

### V. Tools of Continuous Performance Improvement (CPI)

As employees of Seattle Children’s, OAC staff were briefly introduced to CPI methodology [20], [21], [24] on day 1 of their employment. Subsequently, OAC staff were varyingly exposed to CPI, the TPS and the 14 Principles (Table 1). The Research CPI consultant therefore accelerated the OAC’s exposure to CPI through daily coaching using Toyota’s 4P Model [7]: Philosophy: It is the view of the Research Institute that the OAC, in its role of supporting research into cures for pediatric disease, adds value to patients of Seattle Children’s Hospital. People and Partners: The OAC, the OAC CPI team, and the Institute’s administrative services engaged with each in order to become a learning organization. Process: Elimination of waste in the OAC was accomplished by applying CPI tools and principles such as reliable methods Footnote S9, standard work (reliable method plus time), 5S (a visually oriented system for organizing shared workspaces) and a daily management system (DMS) that includes WIP visibility boards. Problem Solving: The CPI toolbox to solve problems within the OAC grew rapidly to a current catalog of A3 Problem Solving Reports [27], root cause analysis algorithms, 5S organizing systems, a problem solving board, a DMS, and heijunka boards (see Table 2 for definitions of these boards).

The authors and OAC staff underwent “3 Actuals Walks” in order see OAC processes and to collect current condition data. These walks referred to (i) going to the **A**ctual Place, (ii) talking to the **A**ctual People working in the process, and (iii) observing the **A**ctual Process. Distances traveled, captured with a measuring wheel (Rolatape MM-12, Watseka, IL), were recorded on standard work analysis (spaghetti diagram) forms in the context of how people, materials and communications flowed in OAC processes. Cycle times, measured with a stop watch, were captured on timed observation forms. Identifiable waste was recorded on waste worksheet forms. Reliable methods were consciously developed by the OAC and owned by the OAC CPI team. Standard work documents were crafted by associating time with reliable methods.

### VI. Data Collection

#### Metrics

The number of mouse cages changed in each room was recorded each day for 6 months. The count of daily, weekly, monthly, quarterly and as-needed WIP tasks was recorded each day. A labor study was conducted over a 2 week period in which each staff person recorded the amount of time it took to complete their daily tasks.

#### Electronic data entry and analysis of cage WIP

Counts of cages in use and in reserve were electronically captured once each day via data entry to SharePoint Portal Server 2003 lists (Microsoft, Redmond, WA, USA), as centrally administrated by the Information Services department at Seattle Children’s. Data were also visualized through the use of Tableau Desktop (version 7.0.5; Tableau Software, Seattle, WA, USA), a computer graphics and database query system that provides a robust method to visualize data.

#### Ammonia levels and establishment of a biological kanban

Small animal colorimetric ammonia sensors (Pacific Sentry, Redmond, WA, USA) were placed, for 2 hr, in cages housing 5 mice on approved protocols. Ammonia levels were indicated by color exhibited by the sensor in the low (<50%) humidity range at 1–14 days after cage change. Such environmental monitoring is part of the OAC’s standard husbandry practice. Ammonia levels were collected from cages within SPF rooms. Cages housed within non-SPF rooms were not included in this study.

### VII. Implementation of a Daily Management System (DMS) and Development of WIP Boards

The OAC’s DMS and animal care and use program, was designed to be compatible with the *Guide for the Care and Use of Laboratory Animals* [28]. Our program included, at a minimum, effective plans for preventive medicine, monitoring and treatment of disease, surgery and post-operative care, and anesthesia, analgesia and euthanasia. Since parts of each plan were carried out by OAC staff, communication within the OAC during the assessment and treatment of sick animals was important. Likewise, the daily observations of animals for signs of illness, injury or abnormal behavior need to be conducted by trained personnel. Such observations included holidays and weekends, a process in which a single OAC staff member was expected to visit every cage in the facility – a somewhat daunting task that was standardized by the DMS-dependent implementation of reliable methods and standard work through an A3 Problem Solving Report [27]. The OAC judged implementation of its DMS by evaluating the extent that its processes conformed to specifications set forth by the *Guide* [28].

The animal care and use system was dependent on technically-trained employees who were responsible for the 24,000 sq ft vivarium facility. These Research Institute employees were deployed throughout the OAC as follows: 1 full-time veterinarian, 2 full-time veterinary technicians, 2 full-time animal care coordinators, 5 full-time animal technicians and 1 part-time animal technician.

Implementation of the DMS was successful in improving intra-staff communication via daily huddles. Each morning from 11∶00–11∶15 a.m., the OAC followed huddle standard work in order to *(i)* populate and review their WIP board, *(ii)* solve problems using CPI concepts, *(iii)* identify their daily demand for cages, *(iv)* level load their work through the use of a heijunka board (Table 2; Table 1, Principle 4), *(v)* monitor timelines for 5S inventory control projects, and *(vi)* discuss deadlines, healthcare issues and metric assessments [29]–[31].

WIP and heijunka boards were housed in the hallway outside the OAC staff lounge, the site of daily huddles. Early versions of the WIP board were poster-size prints of an Excel 2007 (Microsoft, Redmond, WA, USA) spreadsheet that was re-issued each week. Each day at huddle, OAC staff checked off which tasks had been completed or were expected to be completed that day; the sum total of these categories was termed Daily WIP. Later versions were crafted onto magnetic white boards of varying sizes in a manner that utilized wet erase markers as a template and dry erase markers for daily markup. For heijunka boards, small, green, red or black magnets provided visual cues for the flow of daily animal healthcare in the various housing rooms.

## Results

### I. Problem Statement, Current Condition, Hypothesis and Root Cause Analysis

The problem was that the OAC failed to sustain process improvement gains achieved after the vivarium was relocated into its permanent home in 2006, despite reporting its improvements in 2008 [23]. The current condition Footnote S10 at the beginning of the research project included a whiteboard listing of 47 tasks created by the OAC. The listing was separated into the following 3 categories: other/daily (n = 9), weekly and monthly tasks (n = 19), and weekly and monthly cleaning (n = 19). While this listing was informative, it was a poor visual design. Mechanistically, it was not a WIP board because it failed to count the work each workday. There was also considerable overlap in tasks listed, as well as inconsistency in the complexity of tasks listed. The collective data indicated that there were ample opportunities to remove waste from these processes.

The goal of this study was therefore to test the hypothesis that reintroduction of CPI tools and principles would facilitate the development and sustainment of a WIP board and a DMS. The project’s targets were designed to align with Seattle Children’s key metric categories: quality, cost, delivery, safety and engagement.

A root cause analysis Footnote S11 of the problem revealed two underlying reasons for the OAC’s lack of CPI sustainment. The OAC had underperformed in *(i)* the refinement of the use of visual controls so that problems would not remain hidden (Principle 7, Table 1) and *(ii)* the growth and development of internal leaders who understood CPI and could teach it to others (Principle 9, Table 1). The PDCA rapid improvement cycle (see Methods) was the principal method utilized to drive CPI-dependent change.

### II. Iterative Improvement #1: Development of a “Count-All-Work” WIP Board

**Figure 1:**
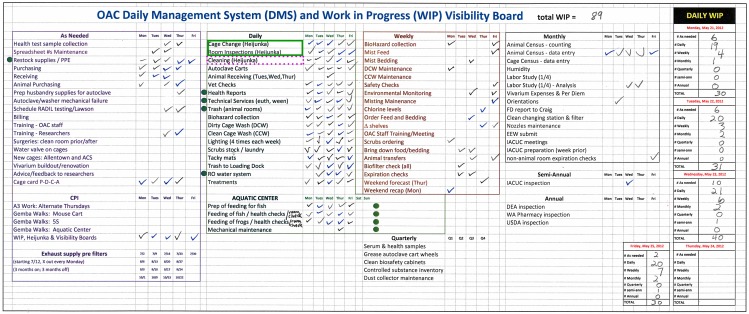
Development of a Daily Management System and a Count-All-Work WIP board for the Office of Animal Care. The Count-All-Work WIP board contained the following 7 categories: As Needed, Daily, Weekly, Monthly, Quarterly, Semi-Annual and Annual. Tasks associated with Cage Changing and Room Inspections (green box in Daily section) were controlled by a separate heijunka board (Figure 2). Similarly, tasks associated with Cleaning (purple dotted-line box in Daily section were controlled by a separate heijunka board (Figure S1). Green filled circles, tasks to be completed during weekends. Representative Daily WIP ranged from 28–40. Representative total WIP = 89.

The 3-category listing of vivarium tasks (above) was translated into a spreadsheet so that OAC staff could visualize and count their work. Over a 3 month period, the amounts and types of work identified increased from 47 to 89–93 and were distributed among 7 time-related categories (Figure 1), an indication that hidden work had been uncovered and made visual. The sum total of each type of work was termed Daily WIP (rightmost columns, Figure 1).

**Figure 2:**
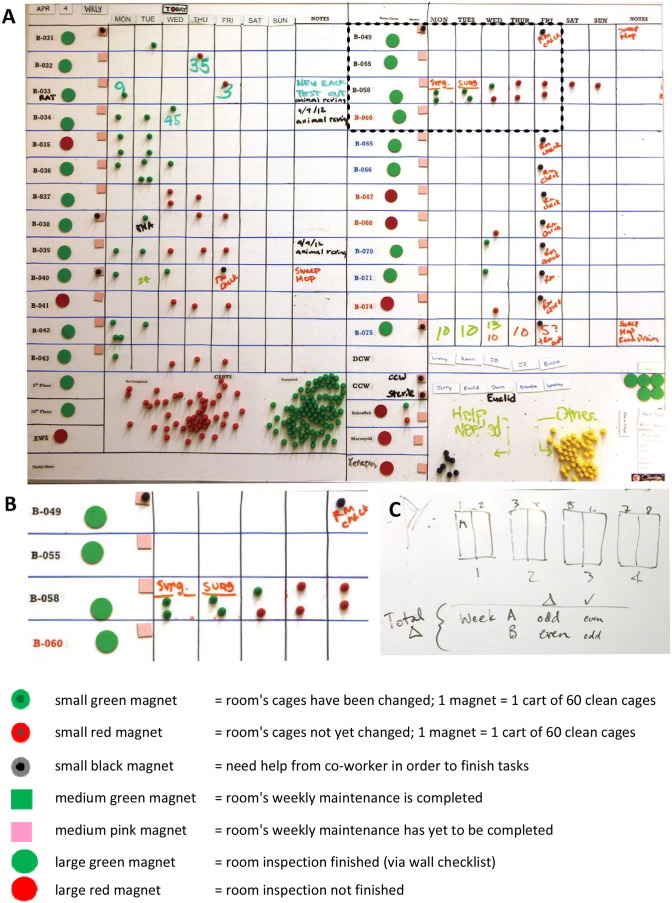
Heijunka (A,B) and side (C) white boards used at daily OAC huddles to level load manpower for processes that involve animal cage changing and room inspection. The OAC’s heijunka boards are the principal focus of daily huddles, and are intended to ensure the completion of daily cage changes and room inspections in a manner that allows the vivarium management to level-load such duties. Small, green, red or black magnets provide visual cues for the flow of daily animal healthcare in the various housing rooms. Panel **B** is an inset from the black-boxed region at the top of panel A. A side white board (Panel **C**) provided a visual aid to guide workers in the original plan of which side of the cage rack to change out each week. In each mouse room, 4 two-sided racks hold a maximum of 120 cages each.

Since the primary function of the vivarium is animal husbandry, tasks associated with this responsibility were called out in the Daily Tasks “Cage Change” and “Room Checks” (green box at top of Figure 1). Given the complexity and importance of these tasks, their scheduling was addressed on a heijunka whiteboard (Figure 2A–B). Tasks associated with weekly cleaning (purple dotted-line box at top of Figure 1) were controlled by a second heijunka board (Figure S1). Coordination and level-loading of these tasks were controlled by this board in a manner that provided staff an opportunity to review the timely completion of these tasks.

**Figure 3:**
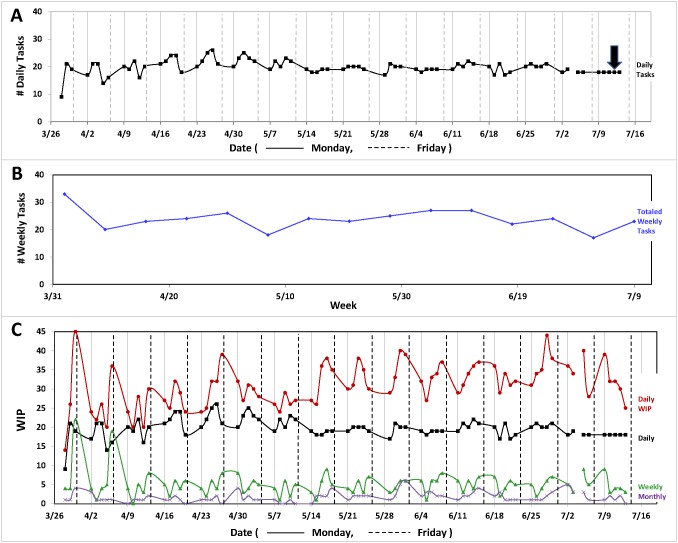
Analysis of the OAC “Count-All-Work” WIP Board reveals staff labor trends in 2012. Data was collected from March 28 through July 13, 2012. (**A**) **Daily tasks.** Solid, vertical line represents Monday. Dashed, vertical line represents Friday. The black arrow designates 100% refinement of what a daily task was and what was not. (**B**) **Totaled weekly tasks.** The sum total of weekly tasks, performed each day, is displayed. (**C**) **Contribution of daily, weekly, monthly tasks to Daily WIP.** The contribution of the Count-All-Work WIP board’s categories (As Needed, Daily, Weekly, Monthly, Quarterly, Semi-Annual and Annual) to daily WIP is shown with a red data points. The daily task component of daily WIP is represented by black data points. The weekly and monthly components of daily WIP are described by green and purple data points, respectively. Solid, vertical line represents Monday. Dashed, vertical line represents Friday.

The Daily WIP number in each category was tracked for 3.5 months, at which time the data were graphed in order to visualize labor trends in 93 types of work processes (Figure 3). Based on the fluctuation of daily task numbers (Figure 3A), OAC staff were eventually able to distinguish between what was and what was not a daily task. By the end of the data collection period, OAC staff had refined the list of daily tasks such that the data points became a straight line (arrow, Figure 3A).

Weekly tasks (Figure 3B), in contrast, fluctuated ±8 around a mean of 24 tasks, a result consistent with weekly tasks not being level loaded. Two types of weekly tasks that contributed to the variance were animal transfers, which are dictated by external forces such as collaborations between investigators at the Research Institute and other research centers, and arrival of shipments of supplies, such as feed and bedding, which occur somewhat unpredictably.

The daily total of tasks in each of the 7 categories, depicted as Daily WIP, is displayed in Figure 3C as a function of time. Early on in the data collection period (March 28– July 13, 2012), Daily WIP was typically highest each week on Fridays. This observation was consistent with the vivarium’s state of affairs in March, as staff frequently saved tasks until the end of week, once the bulk of rodent cage changing was completed. As staff transitioned to an alternative algorithm for cage changing frequency (Methods section VI and Results section V), highest Daily WIP numbers were realized earlier, rather than later, in the week (June-July, Figure 3C, red data points).

Although the “Count-All-Work” WIP Board proved to be an efficient way to capture hidden work, it exhibited a flaw because the 92 tasks were not equivalent to each other Footnote S12. We attempted to reconcile these differences by weighting each of the 92 WIP tasks to 20 categories of a labor study (Table S1), where the latter represented one path in how vivarium *per diem* rates were calculated Footnote S13. Because it was not possible to reliably correlate Daily WIP with labor study time, the Count-All-Work WIP board was determined to be ineffective as a long-term managerial tool and was subsequently abandoned. A rapid PDCA cycle was implemented in a manner that created the following new WIP board suitable for testing.

### III. Iterative Improvement #2: Development of a Cage and Tank WIP Board

Since *Mus musculus* (mice) and *Danio rerio* (zebrafish) are the principal species housed in the Research Institute’s vivarium, and since the bulk of OAC staff time is devoted to caring for these animals, it seemed logical to develop a WIP board that would keep track of all cages and tanks. Such a board (not shown) also tracked rats, frogs and cages going through the cleaning process. After 5 days of testing, it was decided that the plethora of calculations (280 each week) required for rollup to the Cage and Tank WIP board resulted in a visibility board that was of little value to OAC staff. Hence, this board was abandoned due to its inherent complexity. Another round of rapid PDCA process improvement cycles was employed in order to generate a new iteration of the OAC WIP board suitable for testing.

### IV. Iterative Improvement #3: Development of a “Mouse Cage” WIP Board

**Figure 4:**
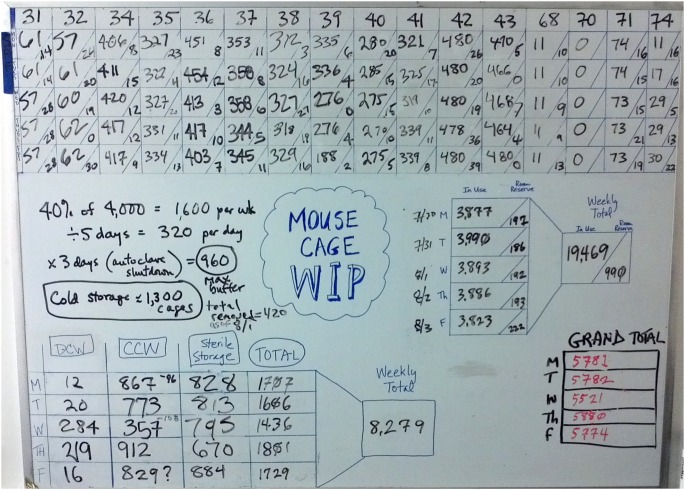
Plethora of calculations required to generate the numbers that rollup to the Mouse Cage WIP Board. In order to reduce complexity and the amount of time spent by OAC staff in reporting out cage and tank numbers to the daily huddle, a board was created to focus exclusively on mouse cages. Staff members were asked to report out the number of cages in each of 16 murine housing rooms, as well as the flow of cages in cleaning processes. Despite the reduction in arithmetic complexity realized by elimination of zebrafish tanks and rat and guinea pig cages, staff were still asked to report out 250 numbers each week to the WIP visibility board (step 2), after first recording the numbers on a scratch sheet in the housing rooms (step 1). A staff member was then assigned to calculate cage sums and report out that total each day to the WIP board (step 3). These numbers were transferred to a spreadsheet via a photograph or recorded onto another sheet of paper (step 4) followed by data entry into a computer spreadsheet (step 5) for analysis. **Top section:** For 16 housing rooms (#31–74, left to right) as a function of week day (top to bottom), the number of cages In Use is the principal value in each box while the number of cages In Reserve is the value to right of the diagonal line. **Center-left section:** Arithmetic calculations that identified 960 clean, sterile cages as the emergency buffer when the vivarium autoclave was out-of-service. **Center-right section:** The daily rollup of WIP numbers for housing room cages In Use and In Reserve. The number of cages listed as Reserve in Rooms indicates a stash of cages made available for the convenience of the researchers. **Bottom-left section:** Daily data for DCW (dirty cage wash), CCW (clean cage wash), Sterile Storage and Total number of cages in the cage cleaning processes. **Bottom-right section:** Grand total number of cages = In Use +In Reserve +Total Cage Wash.

A goal of a Mouse Cage WIP board (Figure 4) was to visualize the daily status of all the mouse cages in the vivarium in a manner that would tell OAC staff “at-a-glance” if additional manpower would be needed. This WIP board, in conjunction with corresponding heijunka boards (Figure 2 and Figure S1), would provide assistance in level-loading animal care assignments, especially when staff members were out sick or on vacation.

Another goal of the Mouse Cage WIP board was to reduce the complexity and the amount of time spent by OAC staff in reporting out cage and tank numbers to the daily huddle. The process by which OAC staff reported out their mouse cage WIP numbers (see legend to Figure 4) was determined to be inefficient because of the 5 steps needed for report outs and to capture the data into a spreadsheet. When it became apparent that multiple users accessing an Excel spreadsheet at the same time was problematic, a reliable method was created that allowed simultaneous data entry into a SharePoint list (Figure S2).

The process measures captured for this improvement are shown in Table 3. Implementation of this reliable method was found to reduce individual OAC employee work from 5 steps to 2. While the lead time for the recording of Daily Cage WIP remained constant at 7.5 hr, the number of handoffs and queues was reduced. With respect to quality levels, there were at least two opportunities for an error to be passed along: the person who hand-calculated the numbers and the person who manually entered the data into an Excel spreadsheet. After improvement, the level of quality was determined to be 3, as these two opportunities for error were eliminated by inspection of the Daily Cage WIP by the OAC work unit.

**Table 3:** Process improvement metrics for Mouse Cage WIP.

Process Measures	Old Process	New Process
Number of Steps per OAC employee	5	2^2^
Lead Time	7.5 hours	7.5 hours
Cycle Time of electronic recording of WIP	-	0.5 minutes
Number of Handoffs	4	0
Number of Check Steps	0	2^3^
Number of Queues	4	1
Level of Quality^1^	1	3

1 The following two definitions of quality were evaluated: *Fitness for Use* via the customer’s assessment and *Conformance to Specifications* via regulations established by the Institutional Animal Care and Use Committee (IACUC) at Seattle Children’s Research Institute and the *Guide for the Care and Use of Laboratory Animals* [28]. The following 5 levels of quality were defined as follows: Level 1, customer inspects; Level 2, company inspects; Level 3, work unit inspects; Level 4, self-inspection; Level 5, mistake proofing. At Seattle Children’s, our customer is the patient/family. At Seattle Children’s Hospital, our customer is evaluated for medical care in our clinics and operating rooms. At the Research Institute, our customer is interacted with only through research protocols approved by the human subjects Institutional Review board (IRB).These interactions are carried out by researchers and monitored, on behalf of the customer, by stakeholders such as researchers, the IRB and external funding agencies (*e.g.* the National Institutes of Health, NIH). In situations where animals are used in research, the customer’s stakeholders are researchers, the NIH, the IACUC, and the Association for Assessment and Accreditation of Laboratory Animal Care (AAALAC). Accordingly, in the context of caring for mice in our vivarium, a level 1 quality inspection could involve the NIH and/or the AAALAC. A level 2 inspection would involve the IRB and/or the IACUC. A level 3 quality inspection would be carried by the OAC in a manner consistent with its daily management system. A level 4 inspection would involve the individual OAC employee (e.g. an animal technician). In order to achieve level 5, the OAC’s daily management system and WIP boards would prevent any errors from occurring and ultimately deliver a defect-free product to its stakeholders.

2 The number of steps for each OAC employee is equal to 2. One person from the team then rolls up the numbers to a Visibility white board.

3 SharePoint 2003 lists, through its inherent Access database, provide mistake-proof mathematical operations of each OAC employee’s WIP numbers. The OAC team, at its daily huddle, provides the 2^nd^ check step.

The Mouse Cage WIP Board also contained daily metrics of cages flowing through a 3-phase cleaning process: dirty cage wash, clean cage wash and sterile storage (Figure 4, bottom-left section). The level of flow continuum for the cage wash processes was determined to be of type supermarket pull (Table 4), where upstream processes replenish what downstream processes took away. The following two types of monuments have restricted the OAC from advancing toward 1 piece flow: *(i)* immovable autoclaves and cage/cart washers, and *(ii)* periodic autoclave breakdowns.

**Table 4:** Flow Continuum Categories.^1^

Traditional Batch & Queue Ideal State of Lean				
**Push or Scheduled**	**Supermarket Pull** **(Kanban)**	**Sequenced Pull** **(broadcast)**	**First-In-First-Out** **(FIFO) Sequenced Flow**	**Continuous Flow** **(1 piece Flow)**
Each process isscheduledand then pushedto the next	An upstream processreplenishes what adownstreamprocess took away	Inventory is pulledfrom a feeder in sequence	Defined lane withStandard Work inProcess betweenunlinked processesin a FIFO sequence	Physically linkprocess steps withno inventorybetween

**Figure 5:**
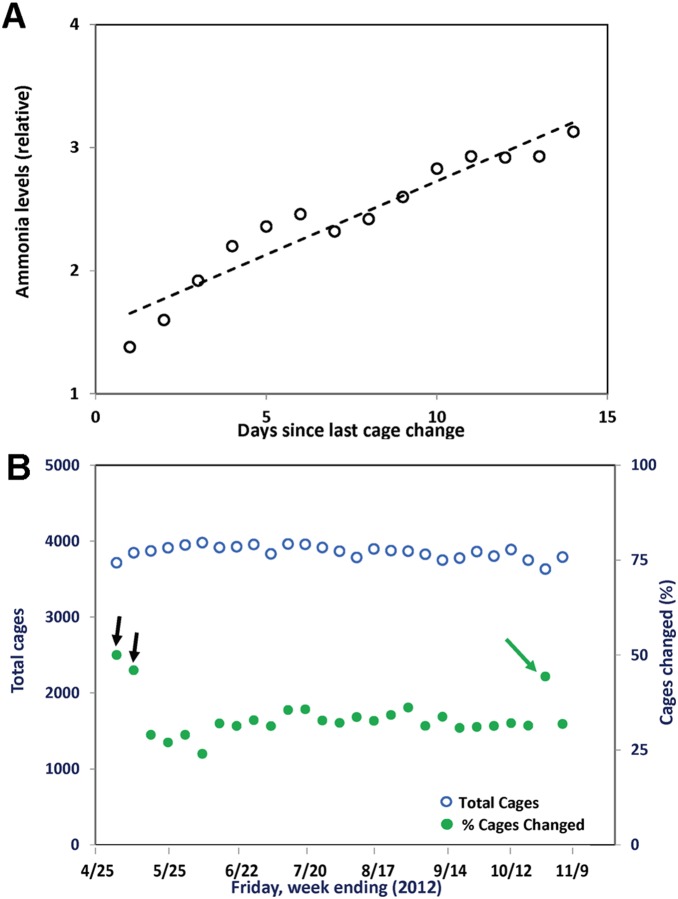
Reduction in the frequency of cage changing as a function of ammonia emissions. (A) Ammonia levels as indicators. Shown are the mean ammonia levels inside cages as a function of time. Cages housing female (n = 5) or male (n = 5) mice were monitored for ammonia levels using an in-cage sensor. Values on the Y-axis are relative levels from the sensors expressed as parts per million (ppm). These data were then correlated with visual inspections of dirty cages. OAC staff were then instructed to change cages based on direct observation of urine and feces patterns. “Low”, “medium” and “high” conditions were observed at relative ammonia levels of 1 (0–1 ppm), 2 (1–25 ppm), and level 3 (25–50 ppm). “Dangerous” conditions (relative level 4, >50 ppm) were not observed. The ammonia level data for males and females on each day were then combined, averaged, and displayed as shown in panel A. The strength of the linear relationship between values for the dataset was evaluated by linear regression analysis. The trendline (dotted line) for the dataset exhibited a coefficient of determination (r^2^) of 0.92, indicating that the regression line fit the data very well. (**B**) **Reduction of % cages changed each week.** Total mouse cages in vivarium during the period indicated are shown by open blue circles. Percentages of cages changed are shown by filled green circles. The pair of black arrows represent cage changing rates prior to ammonia study. The 44% spike at 10/26 (single green arrow) was due to introduction of a new cage type.

1 Abstracted, with permission, from Figure 5–11 of reference [15].

### V. Reduction in Cage Changing Frequency via Implementation of a Kanban Pull System

Prior to April, 2012, vivarium staff attempted to change as many cages as possible on Monday and Tuesday of each week in order to meet the weekly goal of 50% change outs. The main heijunka board (Figure 2) was frequently populated on Mondays with black magnets, as a call for help as staff projected that they would be behind their cage changing by Friday, when most of the weekly tasks were performed. This represented an example of an inventory push system Footnote S14 that forced work into OAC processes based on forecasts, not on actual demand – and violated Principle 3: “use pull systems to avoid overproduction” [34].

After hypothesizing that staff was changing mouse cages too often, a PDCA-based study was initiated to correlate urine spot size and density with cage ammonia levels. The collective data demonstrated that ammonia levels reached 25 ppm (3 on the colorimetric ammonia scale) at day 14 (Figure 5A). When urine spots and fecal patterns reached size and density indicating 25 ppm ammonia, a level that avoids toxicity, OAC staff transferred mice to clean cages with fresh bedding. Such spots and patterns constituted a biological kanban, confirming and extending an earlier report that only focused on spots and patterns [20].

A pull system was subsequently implemented in which the cage changing schedule was solely based on the primary biological kanban system, which directly correlated with demand. After 6 months of data collection, OAC staff calculated that ∼33% of total mouse cages were changed each week (Figure 5B). After a PDCA cycle, the kanban-based cage changing schedule was improved to the present plan, in which dirty cages on both sides of one rack per room are changed on each of 4 days per week per an as-needed basis. This ∼17% reduction in cage changing frequency provided OAC staff with additional time to support Research Institute investigators in their mutual goal of advancing cures to pediatric diseases and conditions.

### VI. Clean Cage Inventory in Housing Rooms: Waste or an Essential Convenience?

Once the OAC achieved a stable process with respect to cage changing frequency, attention was turned to understanding the dynamics of total cage inventory in each housing room. Each morning, OAC staff opened a SharePoint list and entered the number of cages “In Use” and the number of cages “In Reserve” (Figure S2), where the latter count represents clean, sterile cages that were leftover from the quantity that staff pulled from the clean, sterile supply room. Such a quantity typically was defined as a cart of 42 clean, sterile cages. Since each cage represented its own unique environment with respect to experimental parameters, strain, gender, breeders, pups, urine and feces, it was not practical to predict, within the constraints of a reasonable amount of time, with 100% accuracy how many cages were to be pulled in response to the biological kanban.

**Figure 6:**
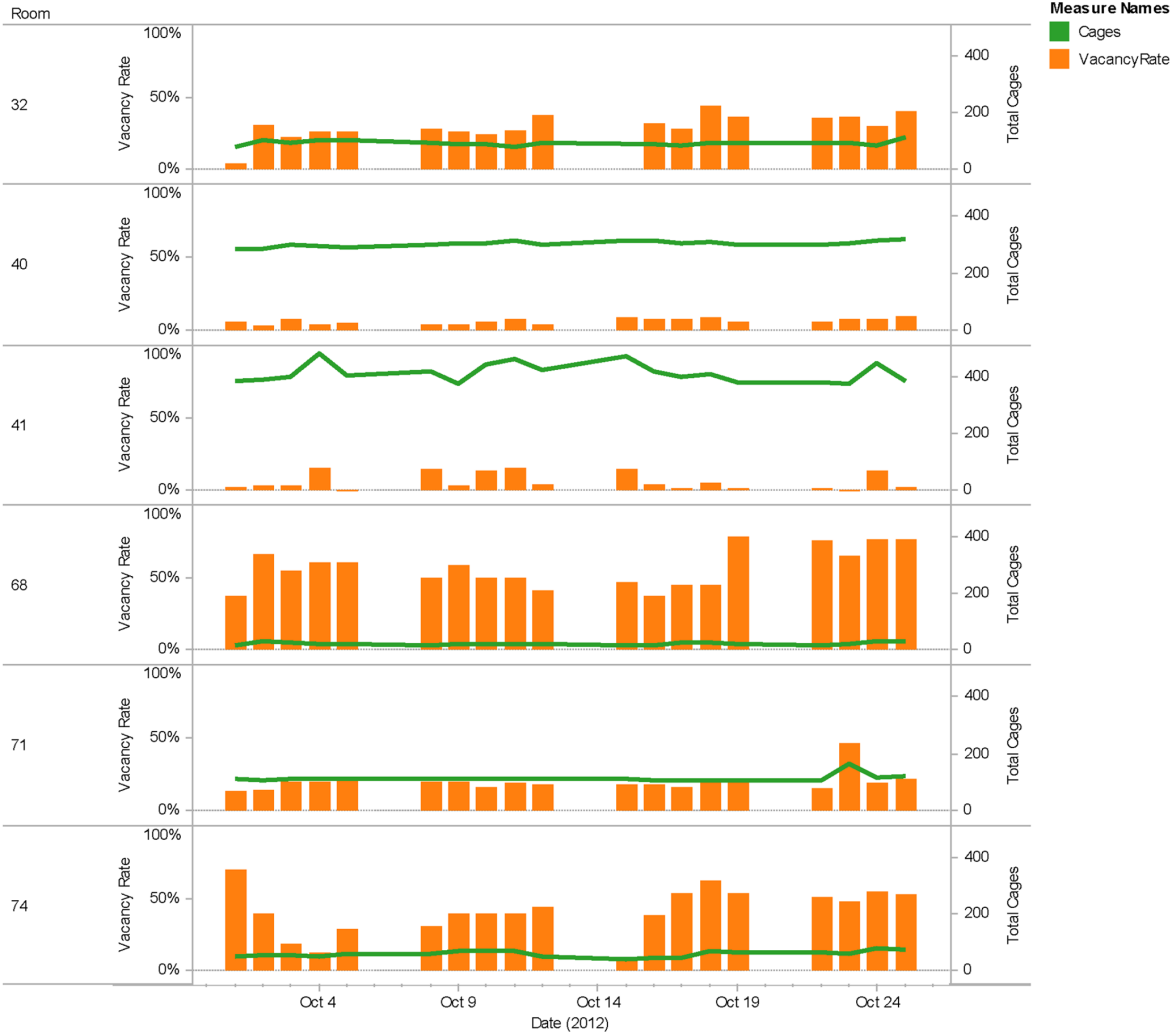
Analysis of Mouse Cage WIP data indicates high rates of vacant cages in housing rooms. Shown are representative data from 6 of 16 murine housing rooms, in which Cage WIP data is displayed in Tableau 7.0.5, for the month of October, 2012. Vacancy Rates, defined as the number of clean unused cages (“In Reserve”) divided by the total number of cages, are shown by orange bars. Total Cages are represented by green lines. While the In Reserve inventory in most rooms was negligible compared to total cages, a few rooms exhibited Vacancy Rates >40%. For a given room in October, there were 17 instances in which # In Reserve ≥ # Total Cages, 45 instances in which # In Reserve >50% In Use cages, and 20 instances in which Vacancy Rates were ≥50%, observations that are probably not unexpected as the bulk of these instances were in quarantine rooms (*e.g.* room 68*)*.

Consequently, there were often clean, sterile cages left over. Since guidelines prohibited the transfer of clean, sterile cages from one housing room to another, there were variable quantities of In Reserve cages left in each room each day. Analysis of the In Reserve inventory for October 2012 is presented in Figure 6 in the context of Vacancy Rates, defined as the number of clean unused cages (In Reserve, orange bars) divided by total number of cages (green lines). The data illustrate how the restriction on moving clean cages from one housing room to another can identify potentially wasteful inventory in the context of the CPI concepts known as flow and pull.

But are these In Reserve cages an example of wasteful inventory? OAC staff maintained that In Reserve clean, sterile cages were left in the room for the convenience of the researcher, who often will conduct experiments requiring additional cages after hours Footnote S15. Could staff find a way to approach 100% accuracy in how many cages were to be pulled in response to the biological kanban? One multi-step scenario would involve staff (i) spending significant, additional time to visit each cage, (ii) calculating the number of cages to be pulled for a given housing room, and (iii) spending significant, additional time traveling back and forth from housing rooms to the sterile cage supply room in order to load one cart with 42 clean, sterile cages. Each trip back and forth would then range 50–320 linear feet (Figure S3), raising the possibility that one type of waste would be substituted for another.

The collective data thus allow the flow continuum assigned to housing room cage inventory to be of type supermarket pull (Table 4), consistent with OAC staff recognizing the biological kanban and subsequently pulling clean, sterile cages. Two types of monuments, defined as immovable objects and processes, have thus restricted the OAC from advancing toward 1 piece flow in housing room inventory: (i) researcher convenience, and (ii) cage movement restrictions akin to the conformance to specification definition of quality.

### VII. Staff Engagement was often Marginal during CPI-dependent Changes

During the 8 months that spanned this project, significant forces aligned themselves to resist CPI-dependent change in the OAC. Countering these forces was the determination of the newly trained Research CPI consultant and the newly hired veterinarian, where the latter served as a model learner. While some staff were excited and engaged, other staff were skeptical about the utility of the CPI exercises and expressed concern that productivity might decline due to the burden of “CPI work”. During this time, the OAC was frequently understaffed, and, after a long day, it just seemed easier for staff to revert to old habits in order to maintain quality animal care and regulatory compliance. Once it became clear that evolution of the OAC WIP board had stalled, the president of the Research Institute personally intervened in order to reset the transition process. Even after the reset, staff remained conflicted over political, technical and cultural aspects of change. After significant contemplation, the veterinarian moved swiftly to implement an OAC-wide reorganization that created several teams, one of which became responsible for championing CPI-dependent change. As a result, progress was accelerated and OAC staff began to respond and align with the messages of CPI. At the time at which this manuscript was submitted, a growing number of OAC staff could explain, through a CPI perspective, what they are doing and why they are doing it. Finally, Principles 9 and 10 (Table 1) had set in and the OAC was able to align their CPI journey with the other departments of the Research Institute.

## Discussion

Research vivaria are at the forefront of biomedical research because of the requirement for pre-clinical animal studies. The IACUC and federal guidelines require vivarium managers to implement and maintain a series of standards that conform to specifications. Many vivaria demonstrate compliance with these requirements via certification by the Association for Assessment and Accreditation of Laboratory Animal Care International and regular IACUC inspections. Accordingly, front line workers perform regular counts of cages and animals in order to establish *per diem* costs and comply with *Guide for the Care and Use of Laboratory Animals* [28]. Such counting is amenable to process improvement through the methods and principles of the TPS.

While the use of “work in process” and “work in progress ” has often been interchangeable in lean methodologies, each of these terms have distinct meanings in the construction [35] and accounting worlds. In the context of the research vivarium, we propose the following distinctions. From the research laboratory perspective, mice in a cage represent one part of a larger research project designed to address a scientific hypothesis; mice in a cage therefore represents work in progress. From the perspective of a vivarium staff worker, mice in a cage represents part of their daily work (health inspections, weaning, breeding, cage changing); mice in a cage therefore represents work in process.

Sustaining the gains achieved from CPI initiatives has proved to be an accomplishment that, to date, has been a challenge at the Research Institute. Before a complex organization can realize long-term lean successes, it will experience a series of phases in which “big wins” are typically offset by momentum-slowing setbacks. The OAC and the Institutional Review Board (IRB) at the Research Institute are examples of how an errant identification of the research investigator as our customer, facility relocation and employee attrition all combined to deny CPI sustainment of the Research Institute’s early big wins in these two support services [20], [23], [24]. Fortunately, our leadership understands that major change will take a very long time (Principle 1). Despite these inherent obstacles, the reintroduction of CPI through the leadership of SCRI’s first full-time veterinarian has served to identify the path that the OAC must take in order to build itself into a lean research vivarium.

The OAC veterinarian attends monthly meetings of the Research Institute’s administrative leadership in which each department reports out their progress in implementing aspects of the TPS. The level of sustainment for the OAC can be verified by at least 6 criteria. *(i)* The OAC’s DMS and WIP board has been operational (>16 months). The OAC WIP is the focus of what is monitored and measured throughout a PDCA cycle. (ii) The OAC has learned how to measure its demand and to use reliable methods to control uniform and consistent processing procedures. (iii) Standard work has been implemented for OAC’s daily huddle (>16 months), for the dirty cage wash process (>8 months) [36] and for environmental monitoring (>16 months). (iv) Each member of the OAC’s CPI team has completed internal Seattle Children’s CPI courses of either 0.5, 1 or 4 days in duration. (v) A DMS assessment radar chart is now in use with a DMS assessment form in order to track overall progress in achieving a robust, and internally-sustainable system and culture of learning. (vi) A 60% reduction in staff overtime was realized, based on a comparison of data 64 weeks prior to and 64 weeks after the project’s start.

In addition to the purpose of calculating *per diem* charges, OAC staff capture their work labor in order to make their WIP visual so that no problems are hidden (Principle 7). These two captures are tightly linked as using tools of CPI has been viewed as a strategy to reduce costs without compromising quality [20]. Despite CPI-dependent improvements, the Research Institute (as well as most academic research enterprises) continues to subsidize vivarium *per diem* rates in order to stabilize charges incurred by multi-year research projects. One exception is the Center for Comparative Medicine at Massachusetts General Hospital which has implemented a lean approach to vivarium management [37]. The removal of waste and process improvements has converted the Center from operating in a deficit to annually realizing a small profit [38].

The process improvement metrics for Mouse Cage WIP realized a reduction of steps, handoffs and queues. The metrics also realized an increase in check steps and the level of quality. Achievement of quality level 5 was not attained – nor would it be practical, as another OAC staff person would be required to validate the number of cages in a given room. Quality, along with cost, delivery, safety and engagement, are the key metric categories for Seattle Children’s (Hospital, Research Institute, and Foundation). Built-In-Quality and Just-In-Time are the two pillars of Seattle Children’s CPI management system.

While the processes at work in individual housing rooms were out of the project’s scope, the flow of cages through the vivarium was not. The calculation of vacancy rates, defined as the number of clean unused cages (“In Reserve”) divided by the total number of cages, uncovered excess inventories in several rooms. Left in the room for the convenience of the researcher, these cages represent wasteful inventory and violates Principle 3 (“Use pull systems to avoid overproduction”). Given that In Reserve cages reside on a cart, valuable floor space was occupied and continues to be occupied at the time of this article’s submission. Monuments have therefore contributed directly to observed wastes such as overprocessing, inventory, wait time, complexity and space – and constitute the next issues that the OAC will address.

## Supporting Information

Figure S1 **Heijunka board used at daily OAC huddles to level load manpower for processes that involve weekly** **cleaning.**
(PDF)

Figure S2 **Reliable method for report out of daily cage counts based on real-time data entry.**
(PDF)

Figure S3 **Distance traveled between housing and sterile cage supply room.**
(PDF)

Table S1 **Labor study-dependent analysis of “Count-All-Work” WIP.**
(PDF)

Footnote S1 (PDF)

Footnote S2 (PDF)

Footnote S3 (PDF)

Footnote S4 (PDF)

Footnote S5 (PDF)

Footnote S6 (PDF)

Footnote S7 (PDF)

Footnote S8 (PDF)

Footnote S9 (PDF)

Footnote S10 (PDF)

Footnote S11 (PDF)

Footnote S12 (PDF)

Footnote S13 (PDF)

Footnote S14 (PDF)

Footnote S15 (PDF)
