# Supplementary material for: Iterative Development of Visual Control Systems in a Research Vivarium
Source: PLoS One. 2014 Apr 15;9(4):e90076. doi: 10.1371/journal.pone.0090076 (PMC3987998; doi:10.1371/journal.pone.0090076)
Supplement: Figure S1 — Heijunka board used at daily OAC huddles to level load manpower for processes that involve weekly cleaning. (PDF) [file pone.0090076.s001.pdf]

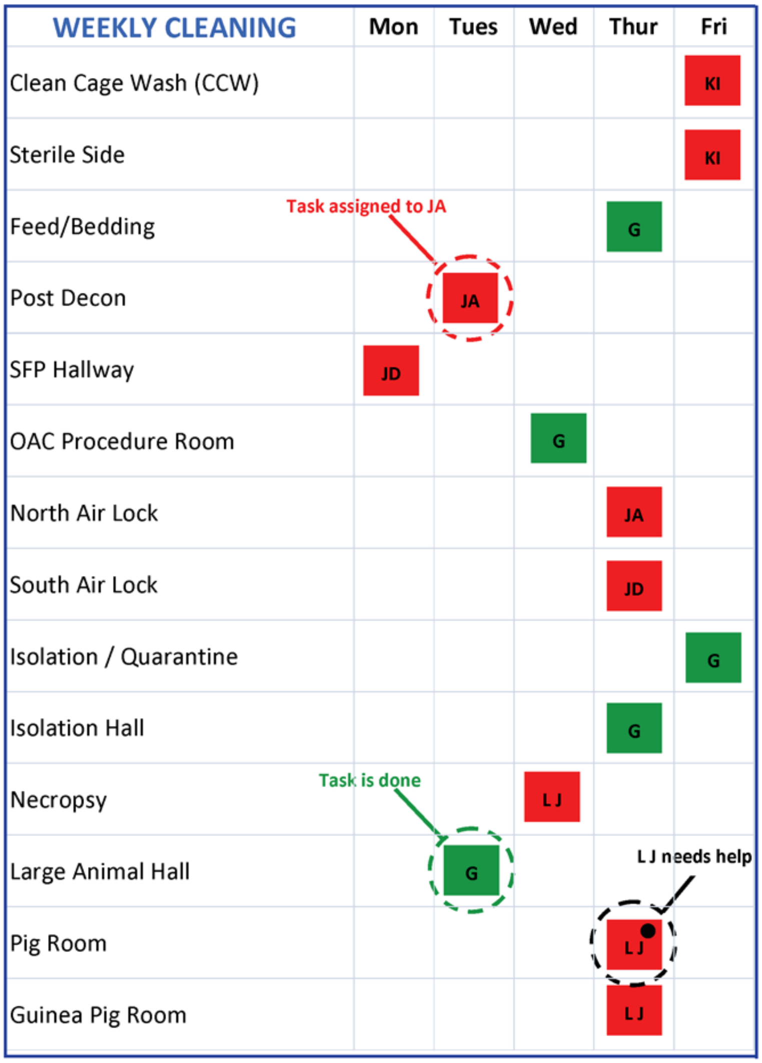

Figure S1. Heijunka board used at daily OAC huddles to level load manpower for processes that involve weekly cleaning. The red side of a square magnet indicates that the cleaning task has yet to be done. The vivarium staff responsible for specific tasks are identified through their initials on the red side only. Once the task is finished, staff turns the magnet over to green (labeled 'G'). A small black magnet atop the red square signals that help is needed. Board in use since April 9, 2012.
