# Supplementary material for: Iterative Development of Visual Control Systems in a Research Vivarium
Source: PLoS One. 2014 Apr 15;9(4):e90076. doi: 10.1371/journal.pone.0090076 (PMC3987998; doi:10.1371/journal.pone.0090076)
Supplement: Figure S2 — Reliable method for report out of daily cage counts based on real-time data entry. (PDF) [file pone.0090076.s002.pdf]

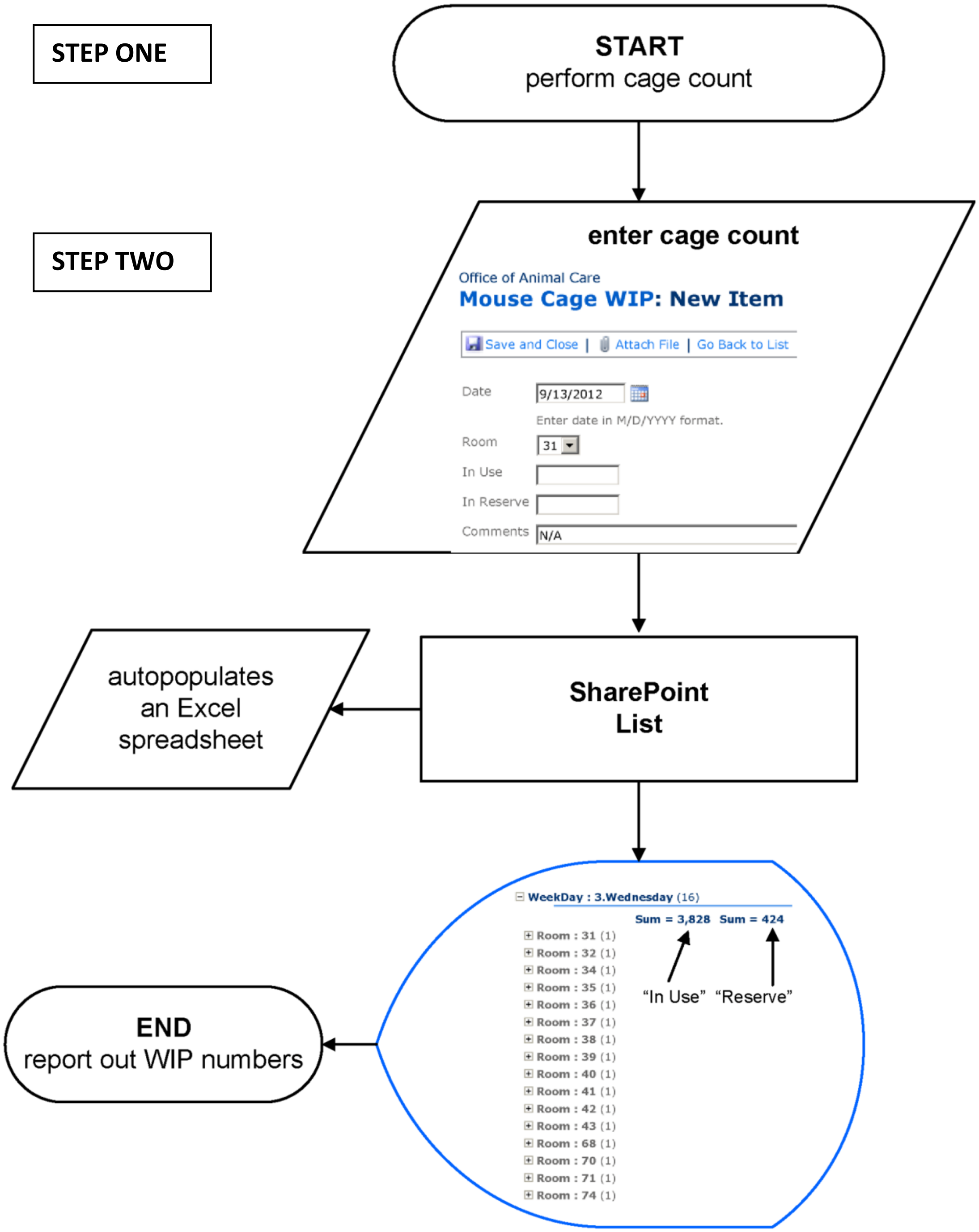

Figure S2. Reliable method for report out of daily cage counts based on real-time data entry.
A SharePoint 2003 list was created to hold data entry of daily cage counts. Drop-down data entry forms were used to select room # and thus provide a mechanism to enter cage numbers. Unlike Excel spreadsheets, where simultaneous data entry by multiple users was problematic, the SharePoint list (via its Access database) proved to be immune from such restrictions. Step 3 (not shown) is not performed by each OAC employee, but instead is rolled up to a Visibility white board by one OAC staff person
