# Supplementary material for: Iterative Development of Visual Control Systems in a Research Vivarium
Source: PLoS One. 2014 Apr 15;9(4):e90076. doi: 10.1371/journal.pone.0090076 (PMC3987998; doi:10.1371/journal.pone.0090076)
Supplement: Figure S3 — Distance traveled between housing and sterile cage supply room. (PDF) [file pone.0090076.s003.pdf]

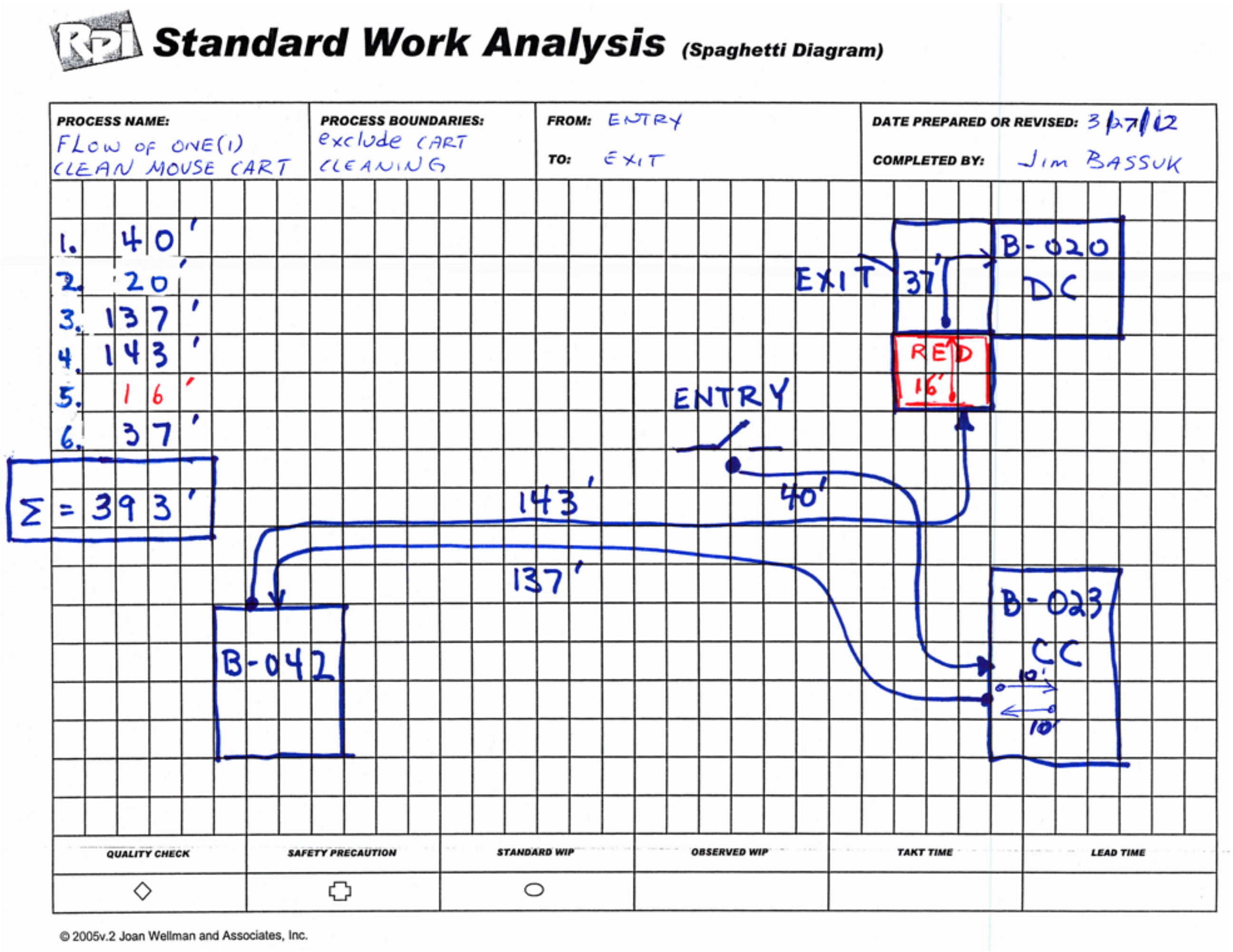

Figure S3. Distance traveled between housing and sterile cage supply room. Shown is a "spaghetti diagram" that illustrates how OAC staff travels from the entry point into the specific pathogen free corridor of the Research Institute vivarium. The animal technician walks from entry to the Clean Cage (CC) room B-023 and pulls 48 cages on a cart. The cart is then wheeled into one of 13 murine housing rooms (shown is B-042). Dirty cages are wheeled on a cart through the Red Zone and then into the Dirty Cage (DC) room B-020.
