# Supplementary material for: Iterative Development of Visual Control Systems in a Research Vivarium
Source: PLoS One. 2014 Apr 15;9(4):e90076. doi: 10.1371/journal.pone.0090076 (PMC3987998; doi:10.1371/journal.pone.0090076)
Supplement: Table S1 — Labor study-dependent analysis of “Count-All-Work” WIP. (PDF) [file pone.0090076.s004.pdf]

Table S1. Labor study-dependent analysis of "Count-All-Work" WIP

Labor Study Category[1]	Labor Study Code[2]	Count of Labor Study Codes from WIP[3]	Daily WIP[4] (%)	Time Spent on Each Labor Study Category[5] (%)
Dirty Cage Wash	A	1	1.1	3.4
Clean Cage Wash	B	3	3.2	4.0
Housekeeping	C	9	9.7	5.2
Animal Receiving	D	1	1.1	0.6
Animal Transfers (in and out)	E	2	2.1	1.2
Animal Purchasing	F	1	1.1	0.6
OAC Training	G	2	2.1	1.1
Animal Health Care	H	8	8.6	6.0
Administrative	I	12	12.9	14.2
Technical Services	J	1	1.1	10.3
Labor Study	K	1	1.1	2.6
Animal Husbandry	L	8	8.6	32.6
Meeting	M	1	1.1	7.7
CPI Activities	N	6	6.4	0.9
Quality Control	O	13	14.0	1.6
Purchasing/Misting/Receiving	P	7	7.5	2.1
Equipment Maintenance	Q	12	12.9	3.1
Researcher Training	R	2	2.2	0.3
Paid Time Off	S	0	0.0	0.9
IACUC	T	3	3.2	1.6
**Total**		**93**	**100.0**	**100.0**

[1] 20 categories from a biannual study by OAC staff during 686 hours over 10 business days in February 2012

[2] Arbitrary codes assigned to each study category

[3] Each of 93 WIP tasks was assigned to a study category; shown are category assignment totals from 7 weeks of data collection (May 7 – June 22, 2012)

[4] ((Count from column 3)/93)x100

[5] ((Hours spent in each study category)/686 hours)x100
