# Supplementary material for: Iterative Development of Visual Control Systems in a Research Vivarium
Source: PLoS One. 2014 Apr 15;9(4):e90076. doi: 10.1371/journal.pone.0090076 (PMC3987998; doi:10.1371/journal.pone.0090076)
Supplement: Footnote S1 — (PDF) [file pone.0090076.s005.pdf]

Footnote S1

On Toyota’s website [4], the TPS is defined as follows: “A production system which is steeped in the philosophy of ‘the complete elimination of all waste’ imbuing all aspect of production in pursuit of the most efficient methods”. The TPS is based on two fundamental concepts: *(i)* Defective products are prevented from reaching the customer and *(ii)* Each process produces only what is needed by the next process in a continuous flow. This latter Just-In-Time concept has been adopted by the National Institutes of Health, the principal research funding agency of the United States government, in which the collection of certain information is postponed until the agency needs it – effectively removing waste from the grant process through reductions in paperwork and record keeping. For a more in-depth analysis of the TPS, the reader is referred to several books on the topic [5–8].
