# Supplementary material for: Iterative Development of Visual Control Systems in a Research Vivarium
Source: PLoS One. 2014 Apr 15;9(4):e90076. doi: 10.1371/journal.pone.0090076 (PMC3987998; doi:10.1371/journal.pone.0090076)
Supplement: Footnote S2 — (PDF) [file pone.0090076.s006.pdf]

Footnote S2

During the late 1980s, the term “lean” was coined by a research team led by Dr. James Womack of the Massachusetts Institute of Technology International Motor Vehicle Program to describe Toyota’s business processes [9].
