# Supplementary material for: Iterative Development of Visual Control Systems in a Research Vivarium
Source: PLoS One. 2014 Apr 15;9(4):e90076. doi: 10.1371/journal.pone.0090076 (PMC3987998; doi:10.1371/journal.pone.0090076)
Supplement: Footnote S3 — (PDF) [file pone.0090076.s007.pdf]

Footnote S3

In 2011, two books were published by CRC/Productivity Press, Taylor & Francis Group. In *Transforming Health Care: Virginia Mason Medical Center's Pursuit of the Perfect Patient Experience* [18], Charles Kenney narrates the establishment of the Virginia Mason Production System and how this organization reset its standards and produced more value for its patients at a lower cost. In *Leading the Lean Healthcare Journey: Driving Culture Change to Increase Value* [19], 40 administrators, clinicians and consultants provide examples of performance improvements over 21 chapters. Between the lines, the reader is able to grasp that Seattle Children's has taken a softer approach to lean, in contrast to the "change or leave" approach of Virginia Mason.
