# Supplementary material for: Iterative Development of Visual Control Systems in a Research Vivarium
Source: PLoS One. 2014 Apr 15;9(4):e90076. doi: 10.1371/journal.pone.0090076 (PMC3987998; doi:10.1371/journal.pone.0090076)
Supplement: Footnote S4 — (PDF) [file pone.0090076.s008.pdf]

Footnote S4

For a given process, there can only be one customer – which can be problematic for research employees not engaged in clinical care to grasp. Early on during the transformation at Seattle Children's Research Institute, the principal investigator was considered to be "the customer" [20]. But if you view the customer as the one who pays the bills, then the Research Institute's customer would be the National Institutes of Health (NIH). But if you ask the NIH who their customer is, they respond with the United States Congress – and ultimately the taxpayer. When you acknowledge that the patient/family is counting on the Institute to provide new cures and treatment for pediatric disease, then the research employee has their "aha" moment in that they now see how their support for research ultimately benefits our true customer – the patient/family.
