# Supplementary material for: Iterative Development of Visual Control Systems in a Research Vivarium
Source: PLoS One. 2014 Apr 15;9(4):e90076. doi: 10.1371/journal.pone.0090076 (PMC3987998; doi:10.1371/journal.pone.0090076)
Supplement: Footnote S5 — (PDF) [file pone.0090076.s009.pdf]

Footnote S5

A stakeholder, in this context, represents the best interests of the customer. At Seattle Children's, the customer is the patient/family. Our numerous stakeholders include researchers, the National Institutes of Health (NIH), the National Science Foundation (NSF), the Bill and Melinda Gates Foundation, the State of Washington Department of Health, the Institutional Animal Care and Use Committee (IACUC) and the Institutional Review Board (IRB).
