# Supplementary material for: Iterative Development of Visual Control Systems in a Research Vivarium
Source: PLoS One. 2014 Apr 15;9(4):e90076. doi: 10.1371/journal.pone.0090076 (PMC3987998; doi:10.1371/journal.pone.0090076)
Supplement: Footnote S6 — (PDF) [file pone.0090076.s010.pdf]

Footnote S6

In an 1994 article, Spear and Bowen argued that if an organization dedicates itself to mastering the following four rules, it has a better chance of replicating Toyota's DNA – and with that, its performance [25]. Like strands of DNA, these rules *(i)* govern how people perform their jobs, *(ii)* how they interact amongst themselves, *(iii)* how products and services flow and *(iv)* how people identify and address process problems. Our study is intended to convey our vivarium's difficulties involved in converting to a culture of continuous improvement.
