# Supplementary material for: Iterative Development of Visual Control Systems in a Research Vivarium
Source: PLoS One. 2014 Apr 15;9(4):e90076. doi: 10.1371/journal.pone.0090076 (PMC3987998; doi:10.1371/journal.pone.0090076)
Supplement: Footnote S7 — (PDF) [file pone.0090076.s011.pdf]

Footnote S7

The first question asked when applying the Toyota Production System to a process is to ask “what does the customer want from this process?” This defines value [12]. Toyota lists the following three categories for value, from the customer’s perspective: *(i)* value added, *(ii)* non-value added and *(iii)* non-value added but required [27].
