# Supplementary material for: Iterative Development of Visual Control Systems in a Research Vivarium
Source: PLoS One. 2014 Apr 15;9(4):e90076. doi: 10.1371/journal.pone.0090076 (PMC3987998; doi:10.1371/journal.pone.0090076)
Supplement: Footnote S8 — (PDF) [file pone.0090076.s012.pdf]

Footnote S8

The concept of a biological kanban is introduced in Table 2 and supported by experimental data in Figure 5A.
