# Supplementary material for: Iterative Development of Visual Control Systems in a Research Vivarium
Source: PLoS One. 2014 Apr 15;9(4):e90076. doi: 10.1371/journal.pone.0090076 (PMC3987998; doi:10.1371/journal.pone.0090076)
Supplement: Footnote S9 — (PDF) [file pone.0090076.s013.pdf]

Footnote S9

Reliable methods are a set of instructions that are *(i)* consciously developed and documented, *(ii)* owned by someone, *(iii)* always followed by everyone performing the work and *(iv)* the basis for improvement.
