# Supplementary material for: Iterative Development of Visual Control Systems in a Research Vivarium
Source: PLoS One. 2014 Apr 15;9(4):e90076. doi: 10.1371/journal.pone.0090076 (PMC3987998; doi:10.1371/journal.pone.0090076)
Supplement: Footnote S10 — (PDF) [file pone.0090076.s014.pdf]

Footnote S10

CPI considers 3 types of conditions: *current condition* is the state of affairs at the time of process mapping prior to any process improvement; *target condition* represents the state of affairs after implementation of countermeasures based on SMART (specific, measurable, attainable, reasonable and timely) goals; *ideal condition* is how processes would work after all waste had been removed.
