# Supplementary material for: Iterative Development of Visual Control Systems in a Research Vivarium
Source: PLoS One. 2014 Apr 15;9(4):e90076. doi: 10.1371/journal.pone.0090076 (PMC3987998; doi:10.1371/journal.pone.0090076)
Supplement: Footnote S11 — (PDF) [file pone.0090076.s015.pdf]

Footnote S11

In CPI, there are two tools used to drill down to the root cause of a problematic process: a Five Whys analysis [32] and a Ishikawa (fishbone) diagram [33]. These two options provide flexibility, depending on the complexity of the problem.
