# Supplementary material for: Iterative Development of Visual Control Systems in a Research Vivarium
Source: PLoS One. 2014 Apr 15;9(4):e90076. doi: 10.1371/journal.pone.0090076 (PMC3987998; doi:10.1371/journal.pone.0090076)
Supplement: Footnote S12 — (PDF) [file pone.0090076.s016.pdf]

Footnote S12

Foremost was the obvious distinction between changing tacky mats and changing cages, which of course varied tremendously in complexity and in lead time.
