# Supplementary material for: Iterative Development of Visual Control Systems in a Research Vivarium
Source: PLoS One. 2014 Apr 15;9(4):e90076. doi: 10.1371/journal.pone.0090076 (PMC3987998; doi:10.1371/journal.pone.0090076)
Supplement: Footnote S13 — (PDF) [file pone.0090076.s017.pdf]

Footnote S13

While 32.6% of labor study time was due to animal husbandry duties, only 8.6% of WIP tasks were assigned to the same study category. Furthermore, while animal husbandry was ranked #1 in labor study time, animal husbandry was ranked #5 in Daily WIP percentage.
