# Supplementary material for: Iterative Development of Visual Control Systems in a Research Vivarium
Source: PLoS One. 2014 Apr 15;9(4):e90076. doi: 10.1371/journal.pone.0090076 (PMC3987998; doi:10.1371/journal.pone.0090076)
Supplement: Footnote S14 — (PDF) [file pone.0090076.s018.pdf]

Footnote S14

Inventory is one of the 9 types of waste (see Introduction). The opposite of pull (defined in the Introduction) is push, which is defined as a system that forces work based on forecasts or schedules – and not on actual demand. An example of push is when a vendor is willing to sell you abundant quantities of a product; often because their inventory has increased to such an extent that price discounts are offered in an attempt to remove the wasteful inventory. This does not imply that the product is a waste, but instead refers to wasteful costs associated with keeping inventory on the factory floor (*e.g.,* building utilities and maintenance). If manufacturing errors have occurred in building such an inventory, then the vendor will be stuck with an overproduction of defective products.
