# Supplementary material for: Iterative Development of Visual Control Systems in a Research Vivarium
Source: PLoS One. 2014 Apr 15;9(4):e90076. doi: 10.1371/journal.pone.0090076 (PMC3987998; doi:10.1371/journal.pone.0090076)
Supplement: Footnote S15 — (PDF) [file pone.0090076.s019.pdf]

Footnote S15

Such a localized inventory kept researchers from visiting, and potentially contaminating, the sterile supply room, a location that houses the next days clean cage inventory as well as 3 days of emergency buffer inventory. Such a buffer was maintained for days when the autoclave was broken or when its steam-supply system was dismantled for inspection.
